# Comparative physiological and full-length transcriptome analyses reveal the molecular mechanism of melatonin-mediated salt tolerance in okra (*Abelmoschus esculentus* L.)

**DOI:** 10.1186/s12870-021-02957-z

**Published:** 2021-04-15

**Authors:** Yihua Zhan, Tingting Wu, Xuan Zhao, Zhanqi Wang, Yue Chen

**Affiliations:** 1 School of Agriculture and Food Sciences, Zhejiang A&F University, Hangzhou, 311300 Zhejiang China; 2 Key Laboratory of Vector Biology and Pathogen Control of Zhejiang Province, College of Life Sciences, Huzhou University, Huzhou, 313000 China; 3 Institute of Horticulture, Zhejiang Academy of Agriculture Science, Hangzhou, 310021 Zhejiang China

**Keywords:** Okra, Full-length transcriptomes, Illumina RNA-seq, Melatonin, Salt stress

## Abstract

**Background:**

Melatonin, a multifunctional signal molecule, has been reported to play crucial roles in growth and development and stress responses in various plant species. Okra (*Abelmoschus esculentus* L.) is a food crop with extremely high values of nutrition and healthcare. Recent reports have revealed the protective role of melatonin in alleviating salt stress. However, little is known about its regulatory mechanisms in response to salt stress in okra.

**Results:**

In this study, we explored whether exogenous melatonin pretreatment could alleviate salt stress (300 mM NaCl) of okra plants. Results showed that exogenous application of melatonin (50 μM) significantly enhanced plant tolerance to salt stress, as demonstrated by the plant resistant phenotype, as well as by the higher levels of the net photosynthetic rate, chlorophyll fluorescence and chlorophyll content in comparison with nontreated salt-stressed plants. Additionally, melatonin pretreatment remarkably decreased the levels of lipid peroxidation and H_2_O_2_ content and scavenged O_2_^•-^ in melatonin-pretreated plants, which may be attributed to the higher levels of enzyme activities including POD and GR. Moreover, a combination of third- (PacBio) and second-generation (Illumina) sequencing technologies was applied to sequence full-length transcriptomes of okra. A total of 121,360 unigenes was obtained, and the size of transcript lengths ranged from 500 to 6000 bp. Illumina RNA-seq analysis showed that: Comparing with control, 1776, 1063 and 1074 differential expression genes (DEGs) were identified from the three treatments (NaCl, MT50 and MT + NaCl, respectively). These genes were enriched in more than 10 GO terms and 34 KEGG pathways. Nitrogen metabolism, sulfur metabolism, and alanine, aspartate and glutamate metabolism were significantly enriched in all three treatments. Many transcription factors including MYB, WRKY, NAC etc., were also identified as DEGs.

**Conclusions:**

Our preliminary results suggested that melatonin pretreatment enhanced salt tolerance of okra plants for the first time. These data provide the first set of full-length isoforms in okra and more comprehensive insights into the molecular mechanism of melatonin responses to salt stress.

**Supplementary Information:**

The online version contains supplementary material available at 10.1186/s12870-021-02957-z.

## Background

Soil salinization is one of the major abiotic stresses that limit plant growth and development, leading to excessive reduction in global agricultural production [1, 2], especially in arid and semi-arid areas [3, 4]. Currently, 19.5% of the irrigated farmland areas is contaminated by salinity (http://www.plantstress.com/Articles/index.asp). Salt stress increases the accumulation of sodium ions (Na^+^) and inhibits the uptake of potassium ions (K^+^) [5], which interrupts cellular ionic and osmotic balance in plants. The overproduction of reactive oxygen species (ROS) induced by salt stress results in membrane injury and oxidative stress [6, 7]. To resist saline stress, plants have evolved complicated antioxidant system that modulates ROS homeostasis, including antioxidants (e.g., peroxidase (POD), catalase (CAT), superoxide dismutase (SOD), and ascorbic acid) and free radical scavengers.

Melatonin (N-acetyl-5-methoxytryptamine), a pleiotropic and amphiphilic indoleamine molecule, was initially identified and isolated from the bovine pineal gland [8]. It acts as an animal hormone involved in various physiological processes, such as circadian rhythm [9, 10], immunological enhancement [11] and antioxidant activity [12]. Until 1995, melatonin was discovered in vascular plants [13, 14], and subsequently considerable research has revealed its importance in alleviating stress. As an antioxidant and free radical scavenger, exogenous application of melatonin enhanced resistance of plant to biotic and abiotic stresses [15–18], which confers plant stress tolerance by improving photosynthesis and redox homeostasis, enhancing activities of antioxidant enzymes, activating downstream signals and regulating the expression of stress-responsive genes [19–21]. In recent years, many studies have shown that exogenous melatonin treatment enhances salt stress tolerance in a variety of plants [17, 19, 22, 23]. Nevertheless, it is still unknown whether such response of melatonin to salt stress is universal for other plants. Moreover, the molecular mechanism underlying melatonin-mediated salt tolerance remain obscure.

Transcriptomics provides an important basis for systematically revealing gene transcription maps and their regulation, clarifying the molecular mechanism of complex biological traits, and understanding the interaction mechanism between genetic and environmental factors [24, 25]. The second-generation high-throughput sequencing technology is easy to produce many fragments and overlapping groups that can not be spliced, and to lose important information such as variable splicing [26–28]. Recently, there have been a number of studies on transcriptome analysis based on single-molecule real-time sequencing (SMRT) by Pacific Biosciences (PacBio) platform, including the second and third generation sequencing hybrid splicing or the second generation correction of the third generation technology, such as *Salvia miltiorrhiza* [29], *Arabidopsis thaliana* [30], *Moso bamboo* [31].

Okra (*Abelmoschus esculentus* L.), also known as *Lady’s Finger*, is an annual food crop, having large, cleft leaves, white to yellow flowers and pyramidal-oblong capsules. It was naturalized in Africa and now has been widely cultivated around the world. In recent years, okra has received increasing concerns of researchers due to its extremely high values of nutrition and healthcare [32]. However, it is very sensitive to saline stress at seedling stage (Additional file 1: Figure S1). In the present study, to get insight into the molecular mechanism underlying melatonin-mediated salt tolerance, exogenous melatonin was applied on okra seedlings to explore its role in response to salt stress. The response of plant growth, photosynthetic process, ROS accumulation and underlying antioxidant responses under NaCl stress were determined. Moreover, we used hybrid sequencing of PacBio and Illumina to analyze the effects of melatonin on okra exposed to salt stress. This study provides comprehensive insights into the physiological and regulatory mechanisms of melatonin-mediated salt stress tolerance in okra.

## Results

### Exogenous melatonin pretreatment alleviated salt stress of okra plants

**Fig. 1:**
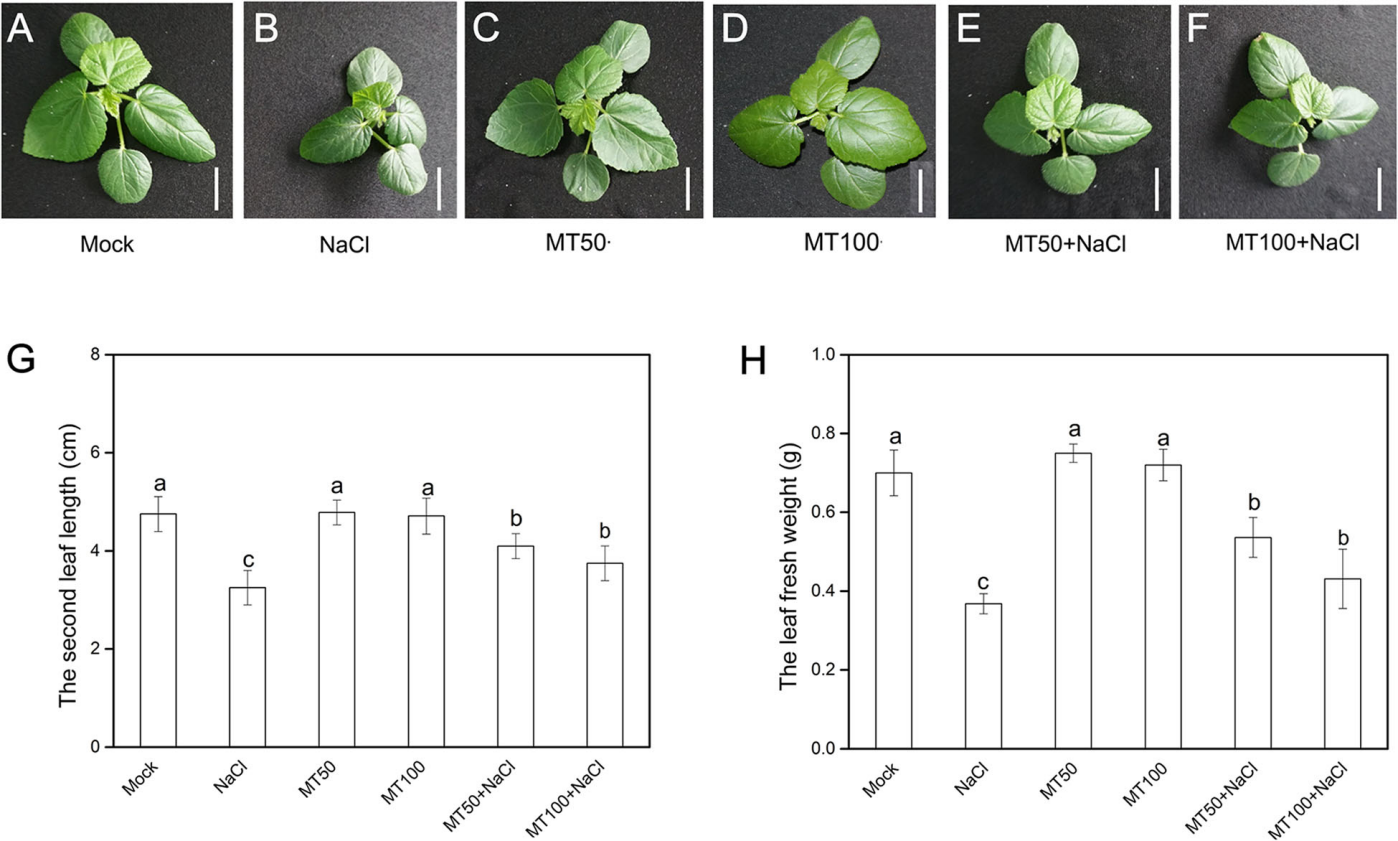
Phenotypic traits of melatonin (50 μM or 100 μM) pretreatment on okra seedlings exposed to salt stress for 7 d by irrigating with 300 mM NaCl, with respective controls. **a-f** Images of leaf phenotype. Bar = 2 cm. **g** The second true leaf length. **h** The leaf fresh weight. Data are means ± SD of six replicates. Bars with different letters are significantly different according to Duncan’s multiple range tests (*P* < .05). Mock, pretreatment with water and grown under optimum conditions; NaCl, pretreatment with water and subsequently subjected to salt stress; MT50, pretreatment with 50 μM melatonin and grown under optimum conditions; MT100, pretreatment with 100 μM melatonin and grown under optimum conditions; MT50 + NaCl, pretreatment with 50 μM melatonin and subsequently subjected to salt stress; MT100 + NaCl, pretreatment with 100 μM melatonin and subsequently subjected to salt stress

To explore whether exogenous melatonin pretreatment can alleviate salt stress of okra plants, melatonin (50 μM or 100 μM) was applied to okra seedlings and the phenotypic traits were observed. The growth of okra seedlings exposed to salt stress (7 days) was inhibited significantly compared with those exposed to normal conditions (Fig. 1a), evident by true leaf dwarfing and deepening in color (Fig. 1b). Moreover, the length of second true leaf and the total leaf fresh weight in salt-stressed plants was 31 and 47% lower than their corresponding mock, respectively (Fig. 1g, h). However, obvious alleviating effects, as manifested by lighter color and bigger leaf, were shown in melatonin-pretreated plants subjected to salt stress (Fig. 1e, f). The melatonin dramatically increased the second true leaf length and the total leaf fresh weight compared with those exposed to salt stress (Fig. 1g, h), and 50 μM of melatonin was more effective than 100 μM. It should be worth noting that phenotypes between melatonin-pretreated seedlings grown in optimum conditions (Fig. 1c, d) and control seedlings (Fig. 1a) have no evident differences, indicating no toxicity symptoms. These results above suggested that melatonin pretreatment can mitigate salinity-induced growth inhibition.

### Effects of melatonin pretreatment on photosynthesis in okra under salt stress

**Fig. 2:**
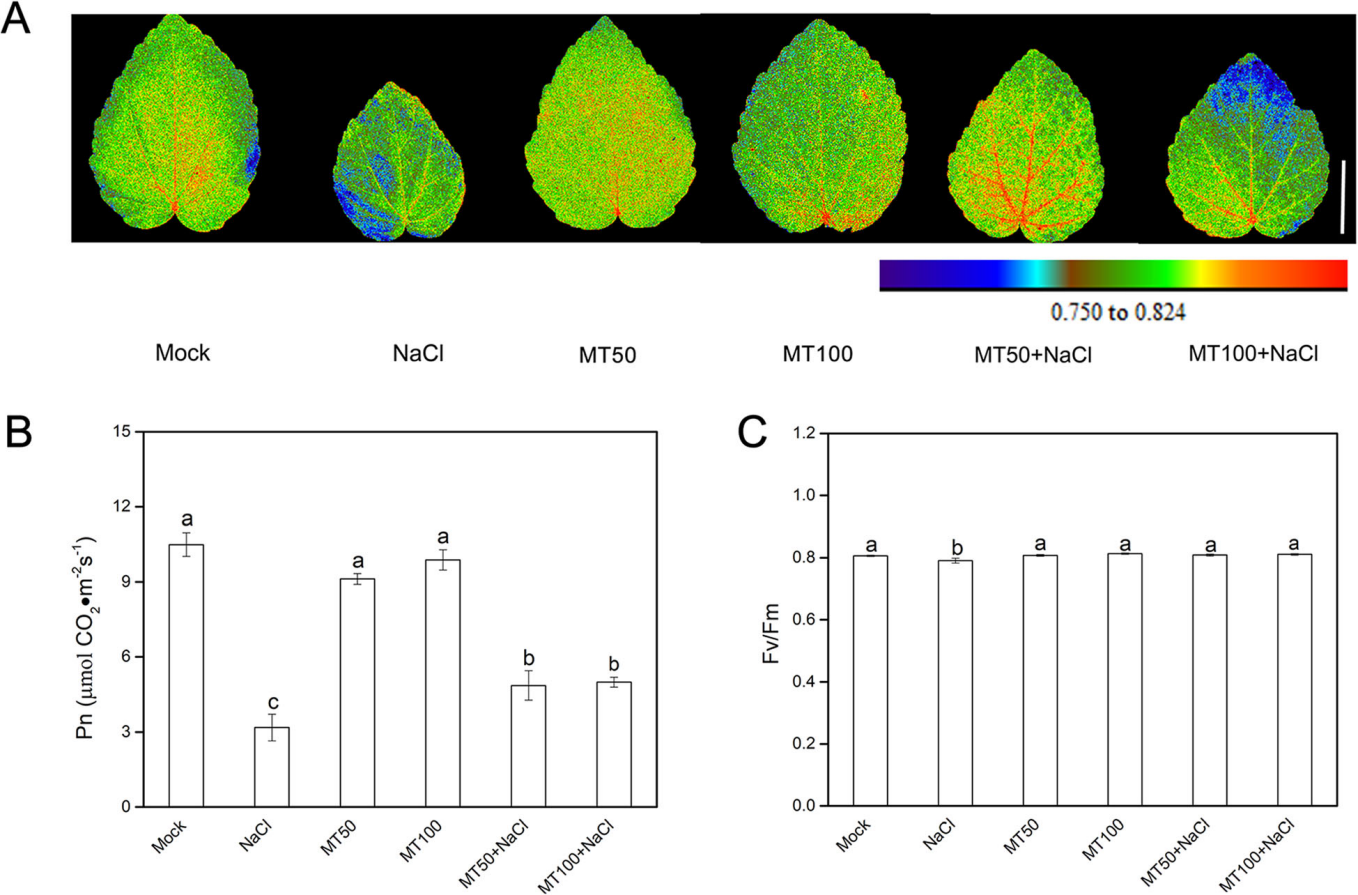
Effects of melatonin (50 μM or 100 μM) pretreatment on photochemical activity and photosynthetic capacity of okra seedlings exposed to salt stress for 7 d by irrigating with 300 mM NaCl, with respective controls. **a** Images of the maximal photochemical efficiency of PSII (Fv/Fm). Bar = 2 cm. **b** Net photosynthetic rate. **c** Fv/Fm values. Data are means ± SD of six replicates. Bars with different letters are significantly different according to Duncan’s multiple range tests (*P* < .05). Mock, pretreatment with water and grown under optimum conditions; NaCl, pretreatment with water and subsequently subjected to salt stress; MT50, pretreatment with 50 μM melatonin and grown under optimum conditions; MT100, pretreatment with 100 μM melatonin and grown under optimum conditions; MT50 + NaCl, pretreatment with 50 μM melatonin and subsequently subjected to salt stress; MT100 + NaCl, pretreatment with 100 μM melatonin and subsequently subjected to salt stress

To further evaluate the effects of melatonin on salt stress responses of okra, the maximum photochemical efficiency of PSII (Fv/Fm) and the net photosynthetic rate (Pn) was monitored. As shown in Fig. 2b, in comparison to mock, NaCl treatment dramatically declined the Pn of okra, accounting for 30.3% of the control. Conversely, pretreatment with melatonin (50 or 100 μM) effectively alleviated the decrease of leaf Pn caused by salt stress. For instance, after subjection to salt stress, Pn in the 50 μM and 100 μM melatonin-pretreated okra plants was fairly higher than those only treated with NaCl, accounting for 46.2 and 47.5% of the control, respectively. Similarly, melatonin pretreatment significantly improved the second true leaf Fv/Fm (Fig. 2a, c). In addition, the Fv/Fm and Pn of melatonin-pretreated seedlings grown in optimum conditions remained almost unchanged (Fig. 2). On the base of both consideration of phenotype and photosynthesis, a 50 μM concentration of melatonin was chosen in the following experiments.

### Effects of melatonin pretreatment on chlorophyll content and oxidative stress in okra under salt stress

**Fig. 3:**
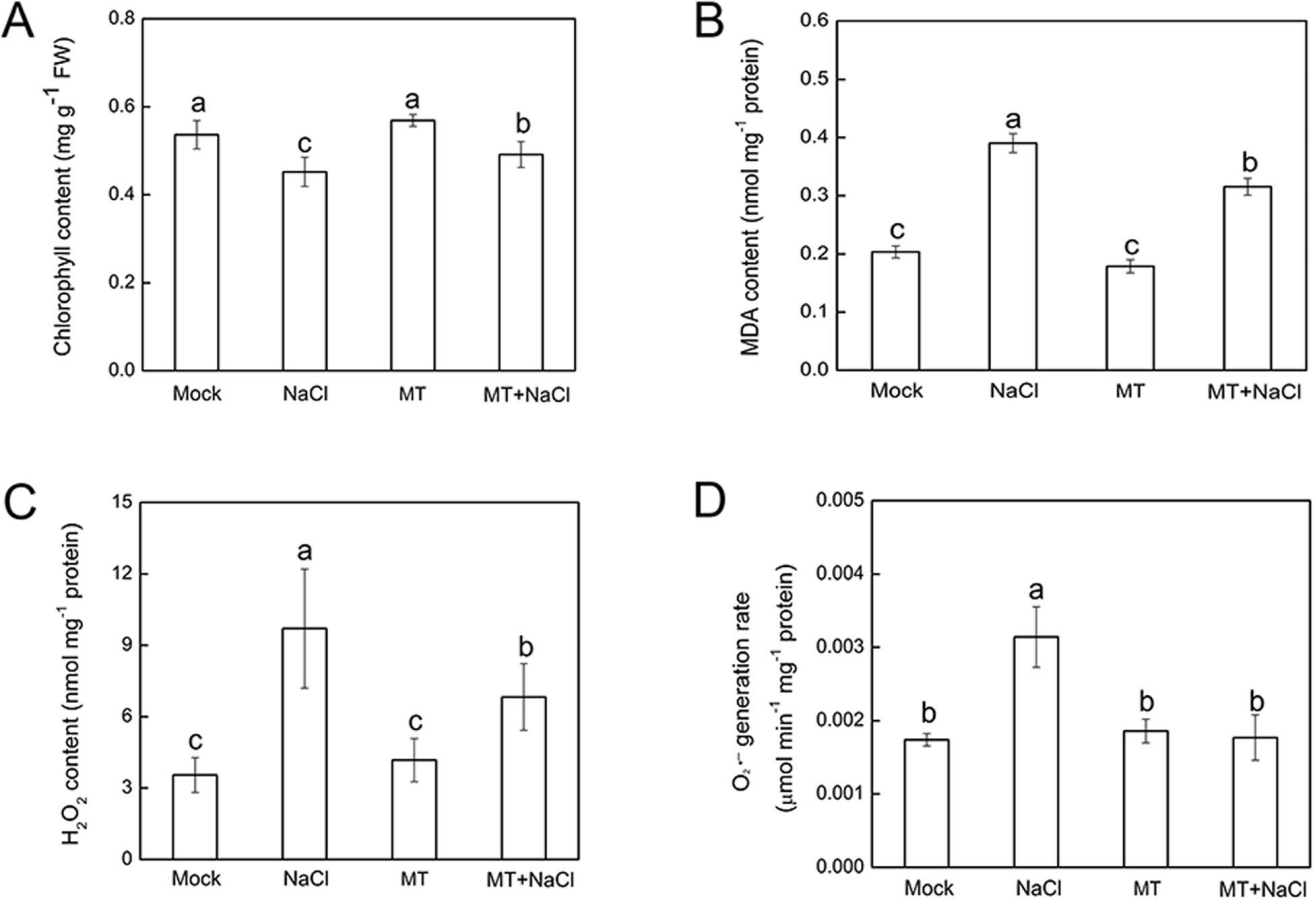
Effects of 50 μM melatonin pretreatment on chlorophyll content, lipid peroxidation, and ROS accumulation in the second leaf of okra exposed for 7 d either to salt stress (by irrigating with 300 mM NaCl) or to optimum conditions. **a** Total chlorophyll content. **b** Malondialdehyde (MDA) content. **c** The content of hydrogen peroxide (H_2_O_2_). **d** The production rate of super oxide (O_2_^•-^). Data are means ± SD of four replicates. Bars with different letters are significantly different according to Duncan’s multiple range tests (*P* < .05). Mock, pretreatment with water and grown under optimum conditions; NaCl, pretreatment with water and subsequently subjected to salt stress; MT, pretreatment with 50 μM melatonin and grown under optimum conditions; MT + NaCl, pretreatment with 50 μM melatonin and subsequently subjected to salt stress

To investigate the salt stress induced oxidative damage and the effect of exogenous melatonin pretreatment on ROS scavenging, the chlorophyll, MDA and H_2_O_2_ contents, and the production rate of O_2_^•-^ were estimated. Total chlorophyll content was declined under salt stress, however, the decline was alleviated by melatonin pretreatment (Fig. 3a). MDA content, the indicator of the degree of cell membrane damage, was significantly higher (2-fold) in salt-stressed plants than that in control plants. In turn, MDA content (1.5-fold of control plants) of melatonin pretreated plants decreased dramatically compared with salt-stressed plants (Fig. 3b). NaCl treatment alone increased the H_2_O_2_ content of leaves by 2.74-fold and the production rate of O_2_^•-^ by 1.8-fold (Fig. 3c, d). However, compared to control, melatonin pretreatment remarkably scavenged the H_2_O_2_ and O_2_^•-^of leaves at 7 days after NaCl treatment, with a 0.82-fold and 0.83-fold decrease, respectively (Fig. 3c, d). Moreover, the total chlorophyll, MDA and H_2_O_2_ contents, and the production rate of O_2_^•-^ of okra plants treated by melatonin itself had no significant difference compared with the control plants, revealing that the melatonin concentration applied was innocuous to plants.

### Effects of melatonin pretreatment on antioxidant enzymatic activities in okra under salt stress

**Fig. 4:**
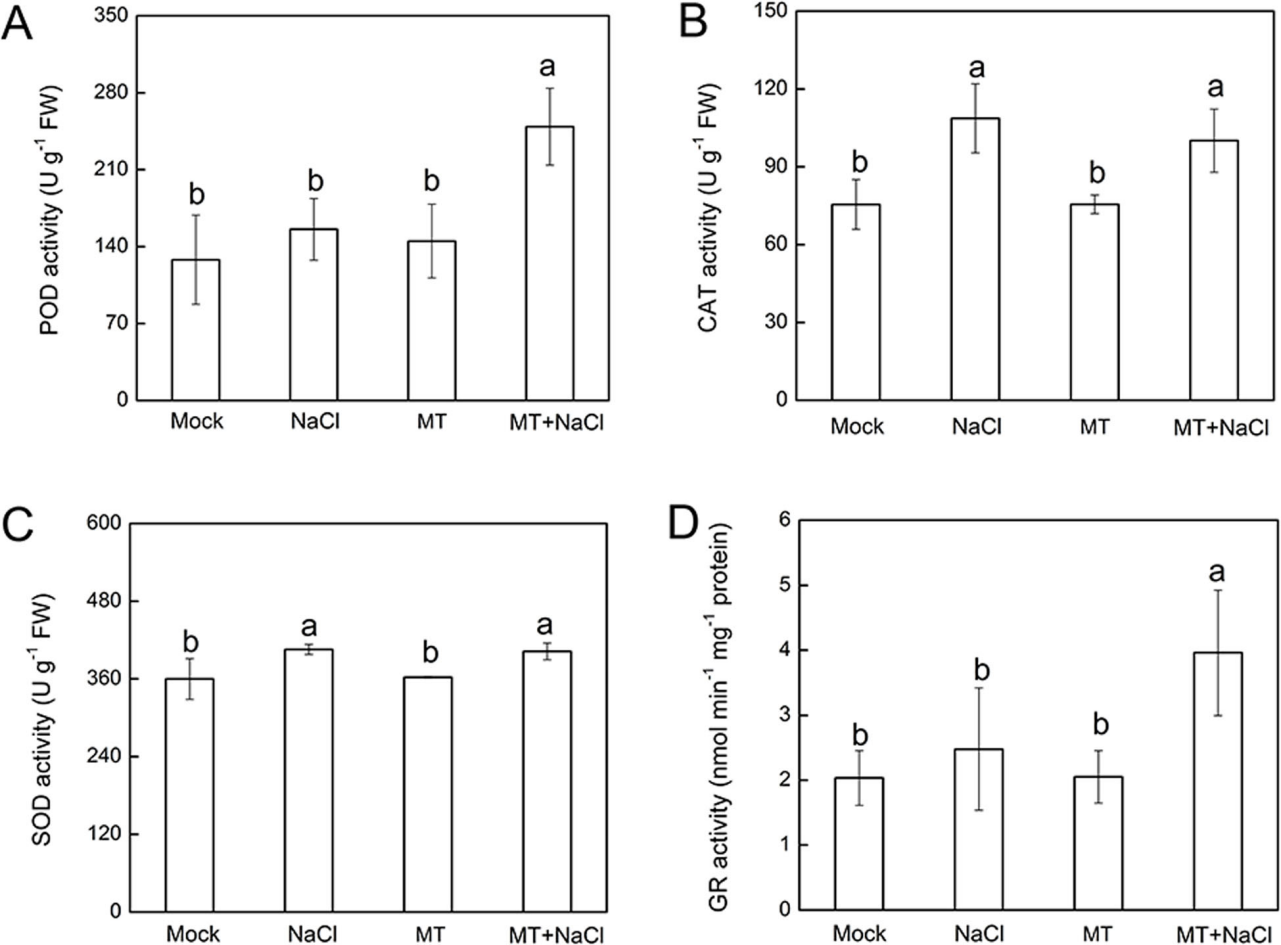
Effects of 50 μM melatonin pretreatment on enzymatic activity of peroxidase (POD) (**a**), catalase (CAT) (**b**), superoxide dismutase (SOD) (**c**), and glutathione reductase (GR) (**d**) in the second leaf of okra exposed for 7 d either to salt stress (by irrigating with 300 mM NaCl) or to optimum conditions. Data are means ± SD of four replicates. Bars with different letters are significantly different according to Duncan’s multiple range tests (*P* < .05). Mock, pretreatment with water and grown under optimum conditions; NaCl, pretreatment with water and subsequently subjected to salt stress; MT, pretreatment with 50 μM melatonin and grown under optimum conditions; MT + NaCl, pretreatment with 50 μM melatonin and subsequently subjected to salt stress

To evaluate whether the decline of ROS accumulation by melatonin was related to the activation of antioxidative system, the activities of POD, CAT, SOD and GR levels were examined. As shown in Fig. 4, salt stress alone significantly increased the activities of both CAT and SOD compared with control plants. Intriguingly, melatonin-pretreated stressed plants showed remarkably higher POD and GR activities (Fig. 4a, d), in comparison with nontreated stressed plants, whereas CAT and SOD activities showed no significant changes among NaCl treatments (Fig. 4b, c). Additionally, the activities of antioxidant enzymes were similar between melatonin-pretreated plants grown in optimum conditions and control seedlings (Fig. 4).

### Okra transcriptome analysis using PacBio sequel platform

**Fig. 5:**
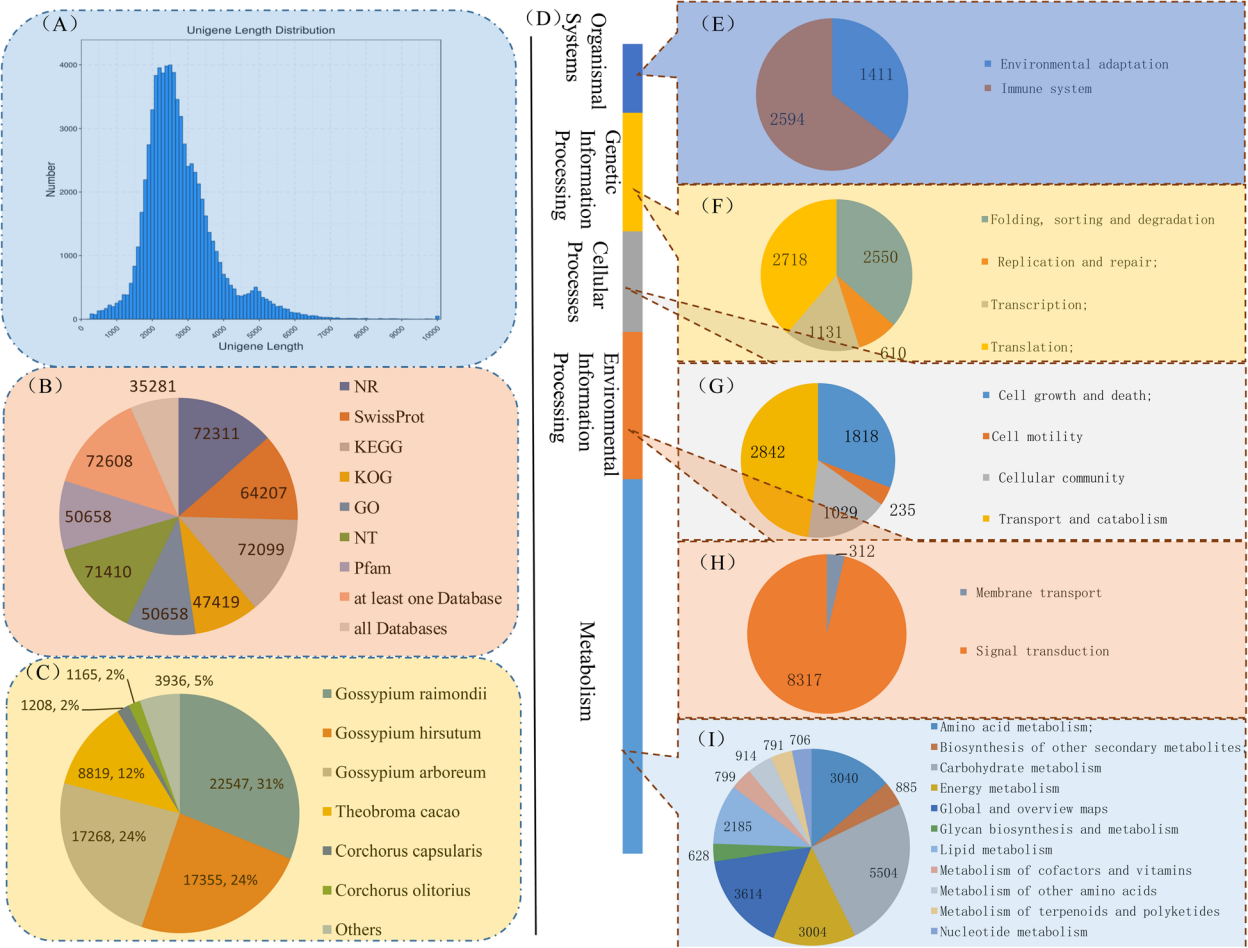
Characteristics of unigenes generated by PacBio sequencing. **a** The length distribution of all assembled unigenes. **b** The number of unigenes annotated by different databases, including NR, NT, Swiss-Prot, KEGG, COG, GO and Pfam. **c** Species distribution of the top BLAST hits for all homologous sequences. **d** A total of 72,099 unigenes were assigned to different KEGG terms. Different color blocks represent different terms, from top to down, “Organismal Systems” (**e**), “Genetic Information Processing” (**f**), “Cellular Processes” (**g**), “Environmental Information Processing” (**h**) and “Metabolism” (**i**). **e-i** Proportion of unigenes in each second level terms in KEGG terms

Total RNA was extracted from three types of okra tissue (roots, stems and leaves). The high-quality RNA was mixed equally for library construction. Libraries were sequenced on the PacBio Sequel platform. Standard Iso-Seq classification and clustering protocol was used to obtain the full transcript sequence. A total of 3,644,038 subreads (8.5 Gb of clean data) were obtained after filtering with SMRTLink (5.1). Then 286,218 circular consensus sequence (CCS) were obtained after the correction between the subreads, of which 221,014 (77.22% reads of total CCS reads) were Full-length non-chimeric reads (Flnc) which contained bar-coded primers and polyA tails (Additional file 2: Table S1). The average subreads length was 2345 bp, and the N50 was 2715 bp. Clustering algorithm and Iterative Clustering for Error Correction (ICE) was used for improving consensus accuracy. Short-read Illumina sequencing was done to quantify the Iso-Seq isoforms by LoRDEC software. CD-HIT software was used to remove redundant and similar sequences by clustering. We obtained 121,360 non-redundant consensus isoforms. The size of transcript lengths ranged from 500 to 6000 bp (Fig. 5a).

### Functional annotation

To analyze comprehensive functional annotation of the 121,360 isoforms, we use BLAST against seven databases (NR, Swissprot, KEGG, KOG, GO, NT and Pfam). A total of 72,608 isoforms were annotated at least one database, and 35,281 isoforms were annotated at all databases (Fig. 5b). Forty-seven thousand four hundred nineteen isoforms were assigned to 25 functional clusters analyzed by KOG database. “General function prediction only” (18.08%, 8574) was the largest category (Additional file 3: Figure S2). We obtained the similarity functional information between sequences of okra and related species by blasting against other plant species via the NR database. The okra isoforms had the highest number of hits to the *Gossypium raimondii* (22,547 hits), followed by *Gossypium hirsutum* (17,355 hits) and *Gossypium arboreum* (17,268 hits) (Fig. 5c), suggesting a high homology between okra and cotton.

To study the function of these genes in biological pathways, the isoforms were identified by the KEGG pathway database. A total of 46,816 isoforms were grouped into five main KEGG functional categories and 213 KEGG pathways (Fig. 5d-i and Additional file 4: Table S2). A high proportion of isoforms were distributed in “Metabolism” (22070) (Fig. 5i), such as “Carbohydrate metabolism” (5504), “Global and overview maps” (3614), “Amino acid metabolism” (3040). To further classify the okra transcripts, GO annotation classification was performed based on three major categories (molecular function, cellular component and biological process) (Additional file 5: Figure S3). For biological process classification, “metabolic process” (24,020 isoforms), “cellular process” (22,818 isoforms) and “single-organism process” (13,849 isoforms) were three major categories. The major category of cellular component was “cells (9294 isoforms)”, “cell parts (9294 isoforms)” and “membrane (6378 isoforms)”. Isoforms involved in the “binding” (33,223 isoforms), “catalytic activity” (24,049 isoforms) and “transporter activity” (2859 isoforms) were highly represented in molecular function subgroups.

### Differential expression gene (DEG) and functional enrichment analysis among different treatments

**Fig. 6:**
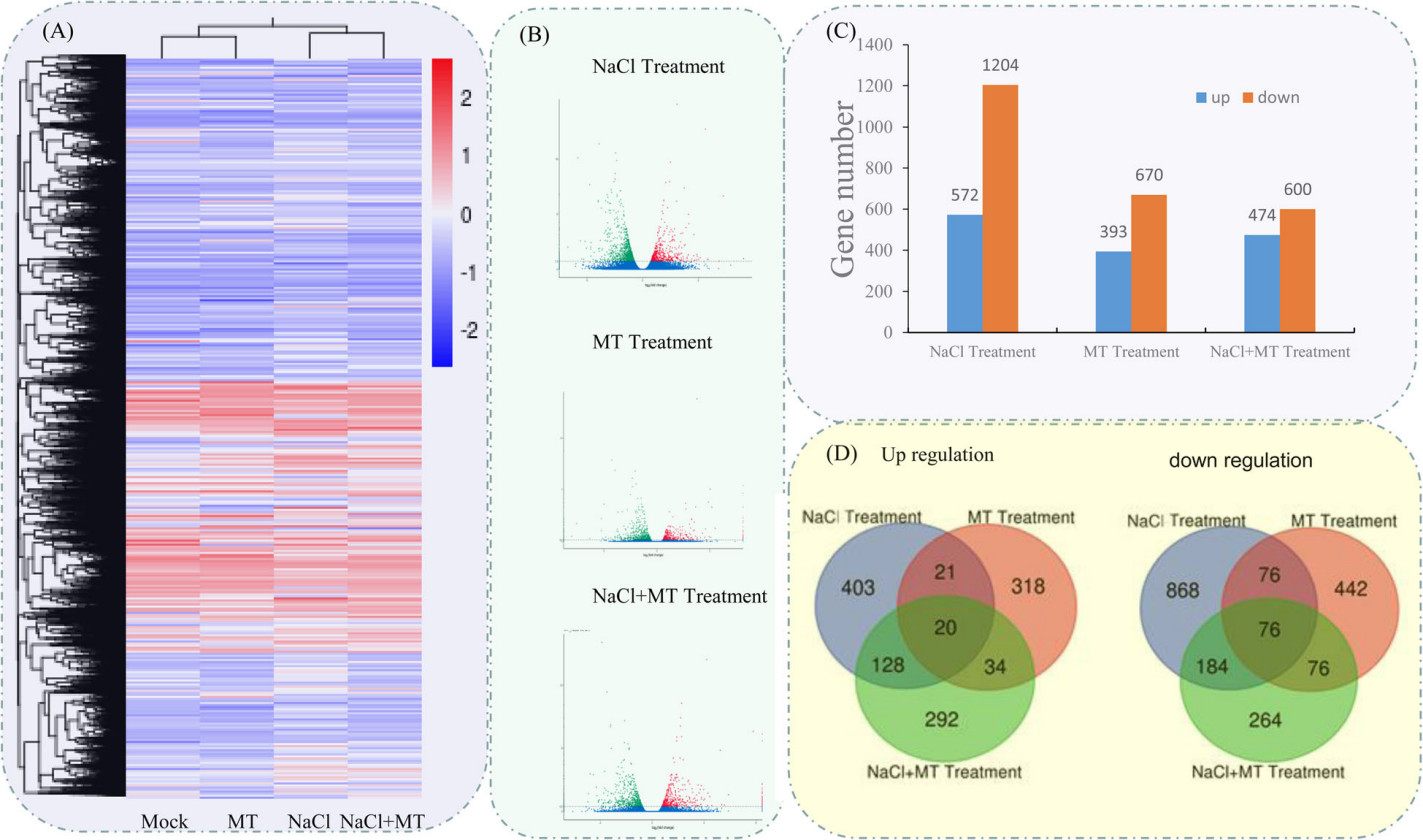
Transcriptional variations in okra shoots under different treatments. **a** Hierarchical clustering analysis of the DEGs under different treatments. **b** Significance analysis of the DEGs in different treatments by Volcano plots. **c** The number of up- and down-regulated genes in different treatments. **d** Venn diagrams showed the proportions of the up- and down-regulated genes in three treatments

To investigate the molecular mechanism underlying melatonin-mediated salt tolerance in okra, the transcriptional expression of the total genes affected by melatonin and NaCl treatments was determined using the Illumina platform. Heatmap of Pearson’s correlation coefficients between all sample pairs showed that each sample was reliable with a good reproducibility (Additional file 6: Figure S4). A total of 551,871,356 raw reads were generated form 12 cDNA libraries (Table 1). Among them, 539,294,676 (97.72%) clean reads were obtained by removing adaptor sequences and filtering low quality reads. We used RSEM software to compare bowtie 2, and further obtained the number of readcounts on each gene in each sample, and then carried out FPKM transformation to analyze the gene expression level. DESeq2 [33] software was used to analyze DEGs with the screening threshold was Padj < 0.05. The input data are readcount data obtained from gene expression level analysis. Cluster analysis was used to determine the expression patterns of DEGs under different experimental conditions. The FPKM values of different genes under different experimental conditions were used for hierarchical clustering analysis (Fig. 6a). The expression trend of these DEGs was showed in Fig. 6b. It turned out that, a total of 1776 DEGs including 572 up- and 1204 down-regulated were identified in NaCl treatment. In MT treatment, a total of 1063 DEGs including 393 up- and 670 down-regulated were identified. A total of 474 DEGs highly expressed in MT + NaCl treatment compared to mock (Fig. 6c). Furthermore, we compared three datasets using Venn diagrams (Fig. 6d). In detail, the DEGs in “NaCl vs. MT”, “NaCl vs. MT+NaCl” and “MT vs. MT+NaCl” groups were determined to be 193, 408 and 206, respectively. There were 96 DEGs common to three comparative groups.

**Table 1:** Illumina-seq output statistics of 12 samples

Sample	Raw Reads	Clean Reads	Clean Bases	Error rate	Q20	Q30	GC content
Mock 1	48,740,818	47,658,684	7.15G	0.03	97.49	92.82	45.04
Mock 2	40,016,058	39,357,798	5.9G	0.02	98.58	95.39	45.28
Mock 3	41,104,166	40,212,232	6.03G	0.02	98.45	95.05	45.23
MT 1	42,853,158	42,126,744	6.32G	0.02	98.49	95.14	44.9
MT 2	43,534,578	42,646,676	6.4G	0.02	98.56	95.35	45.08
MT 3	51,433,522	50,463,954	7.57G	0.03	97.46	92.78	44.89
NaCl 1	47,646,666	46,232,152	6.93G	0.02	98.28	94.68	44.77
NaCl 2	53,066,422	51,991,448	7.8G	0.03	97.37	92.55	44.71
NaCl 3	52,611,268	51,249,888	7.69G	0.03	97.16	92.13	44.98
MT + NaCl 1	41,437,382	39,586,328	5.94G	0.02	98.42	95.01	44.95
MT + NaCl 2	41,476,226	40,860,808	6.13G	0.02	98.46	95.07	45.03
MT + NaCl 3	47,951,092	46,907,964	7.04G	0.02	98.15	94.36	44.89

**Fig. 7:**
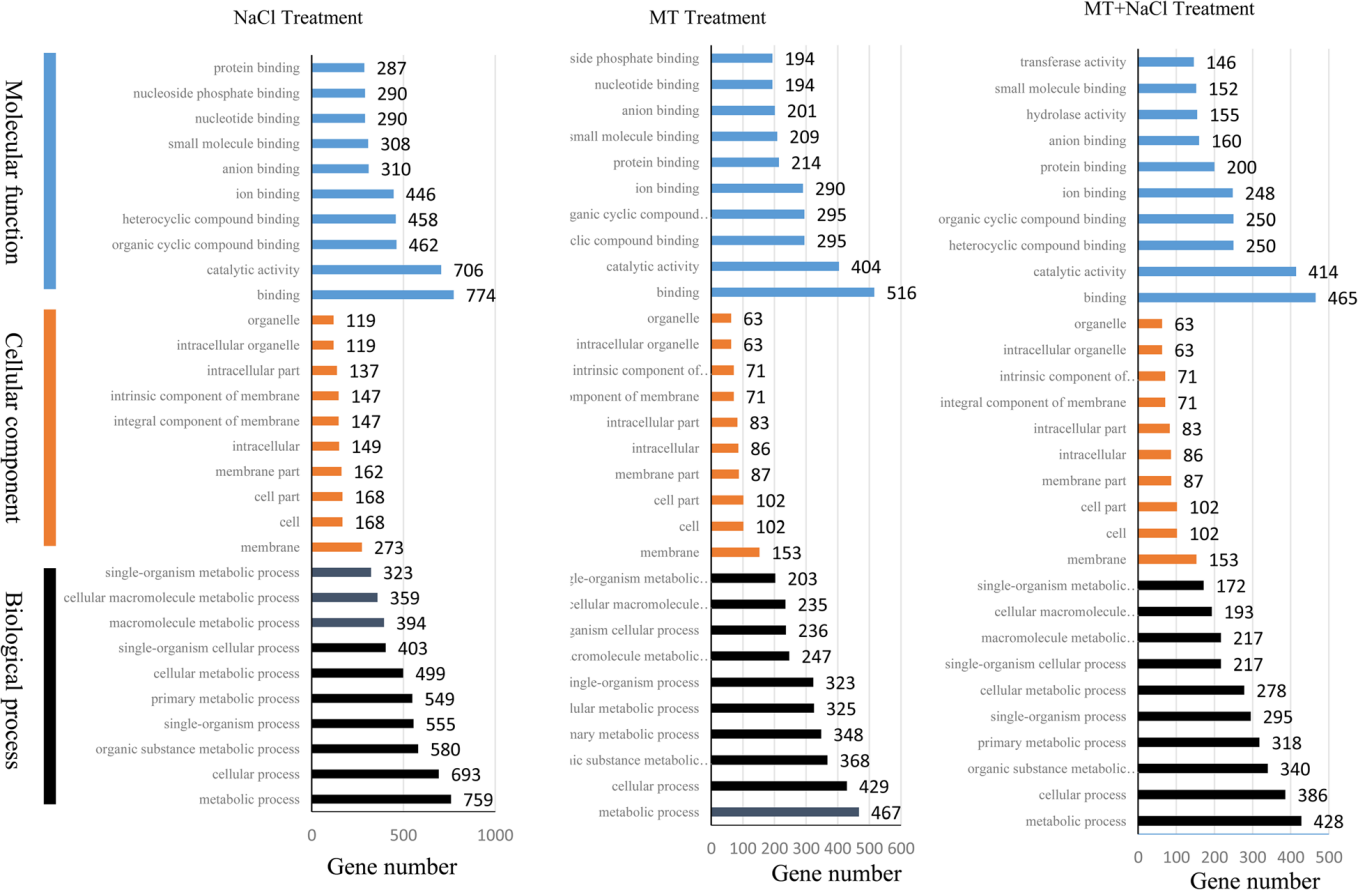
GO enrichment analysis of the DEGs in different treatments. **a** Classification of the enriched GO terms under the NaCl treatment. **b** Classification of the enriched GO terms under the MT treatment. **c** Classification of the enriched GO terms under the MT + NaCl treatment

To elucidate the biological functions of these DEGs, the enriched GO terms were analysed. The top 10 enriched GO terms were showed in Fig. 7. In NaCl treatment, “binding”, “catalytic activity” and “organic cyclic compound binding” were the top three largest GO terms in the molecular function; in the cellular component, “membrane”, “cell” and “cell part” were the top three largest terms; and in the biological process, “metabolic process”, “cellular process” and “organic substance metabolic process” were the top three largest terms. In MT treatment and MT + NaCl treatment, “binding”, “catalytic activity” and “heterocyclic compound binding” were the top three largest GO terms in the molecular function, and the top three largest terms in the cellular component and biological process were the same as those in the NaCl treatment.

**Fig. 8:**
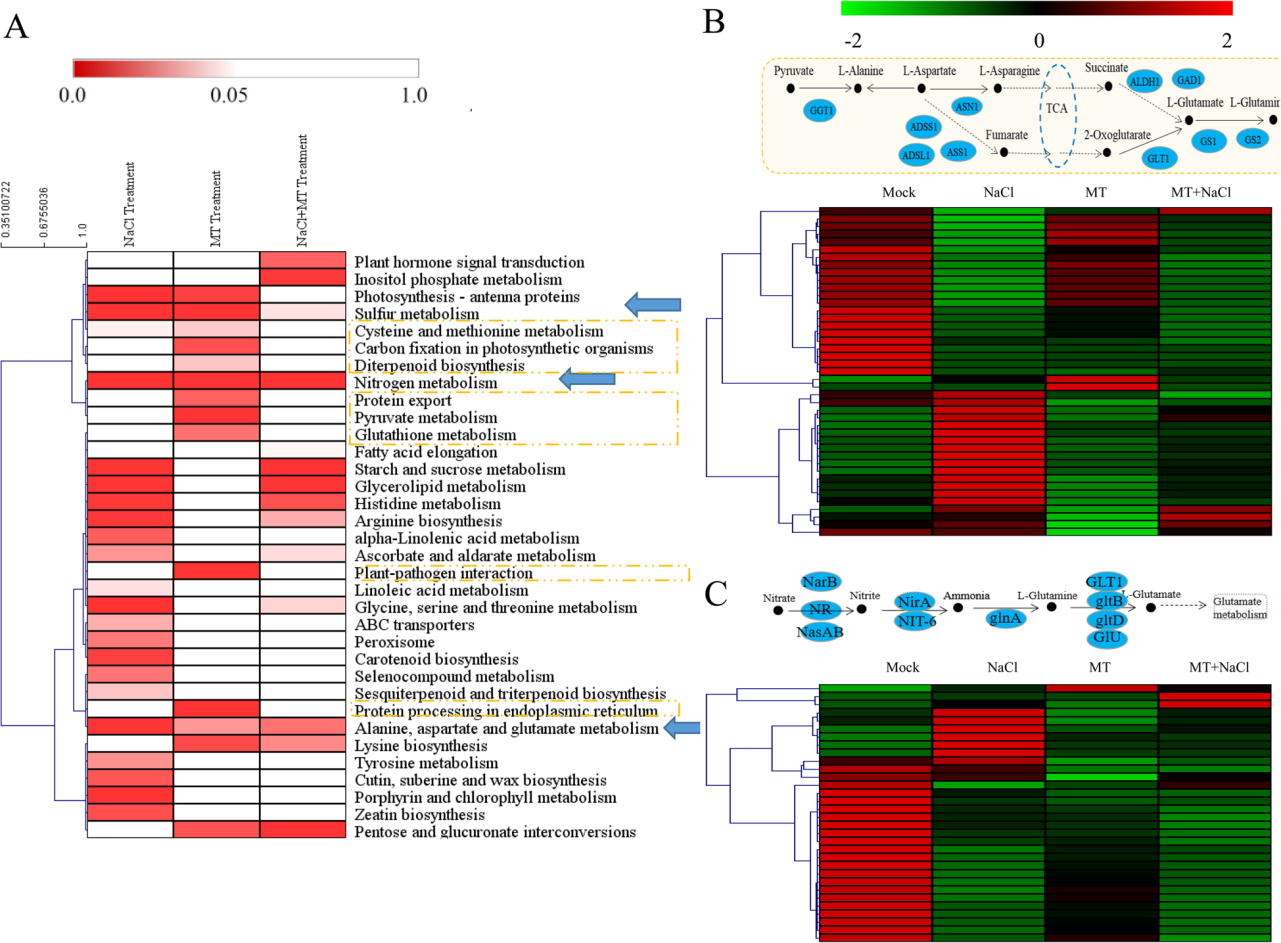
Enriched KEGG pathways analysis of the DEGs in different treatments. **a** The significant *P* values of each KEGG term in three different treatments were shown by a heatmap. Red indicated significantly enriched KEGG terms. Yellow boxes indicated the metabolic pathways only significantly changed under the MT treatment. Blue arrows indicated the pathways significantly changed under all the three treatments. **b** Expression analysis of the unigenes related to the “Alanine, aspartate and glutamate metabolism” pathway. **c** Expression analysis of the unigenes related to the “Nitrogen metabolism” pathway

In total of 34 enriched KEGG pathways (*P* < 0.05) were identified at least in one treatment (Fig. 8a). In NaCl treatment, 23 pathways were significantly enriched. The top three enriched pathways were “Sulfur metabolism”, “Photosynthesis-antenna proteins” and “Nitrogen metabolism”. In MT treatment, 16 enriched KEGG pathways were identified, and “Nitrogen metabolism”, “Sulfur metabolism” and “Protein processing in endoplasmic reticulum” were the top three enriched pathways. In the MT + NaCl treatment, the DEGs were fell in 15 significantly enriched pathways. The top three enriched pathways were “Nitrogen metabolism”, “Pentose and glucuronate interconversions” and “Starch and sucrose metabolism”. Furthermore, “Nitrogen metabolism”, “Sulfur metabolism” and “Alanine, aspartate and glutamate metabolism” were significantly enriched in all three treatments. The DEGs expression profiles of “Nitrogen metabolism” and “Alanine, aspartate and glutamate metabolism” pathways were shown in Fig. 8b and c.

**Fig. 9:**
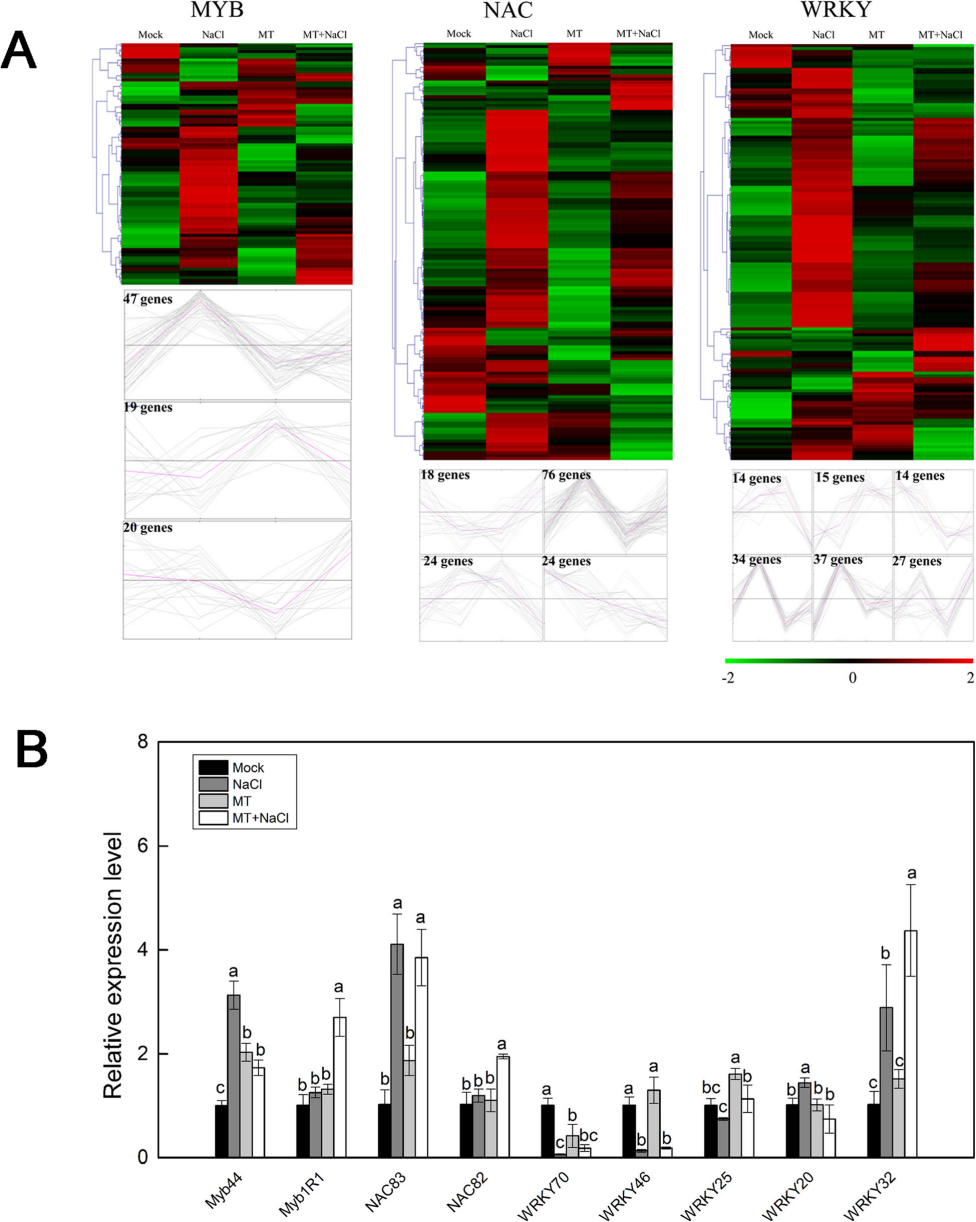
The differential expression pattern of the three TF families (MYB, NAC and WRKY). **a** The differential expression pattern of the three TF families were depicted by a heatmap. **b** Validation of expression pattern of several TF genes by qRT-PCR

Transcription factors play a key role in various aspects of plant growth and development and in the face of adversity stress. We used iTAK software to predict the transcription factors of okra. The numbers of the top 30 largest transcription factor families annotated were showed in a column diagram (Additional file 7: Figure S5). MYB, WRKY and NAC families are the three most studied transcription factor families. The expression profiles of the three TF families in four groups were presented in a heatmap (Fig. 9a). Most of them were up-regulated by NaCl treatment and down-regulated by melatonin treatment. To further verify the RNA-seq data, 9 genes of MYB, WRKY and NAC TF families were randomly selected to perform qRT-PCR (Fig. 9b). The expression patterns of these genes were basically consistent with that of FPKM values by Illumina RNA-seq, which confirms the sequencing data were credible.

## Discussion

*Abelmoschus esculentus* L., an edible medicinal plant, has received more and more attention from researchers, becoming a hot area of research. It contains many kinds of nutrients and important phytochemicals including lectins, flavonoids, glycosides and so on [34]. Moreover, it possesses various biological activities such as antioxidant, antibacterial, anticancer, and anti-inflammatory and immunomodulatory activities [35]. Salt stress, in response to soil salinization, is one of the main factors that limit plant growth and yield. Multiple publications have reported that melatonin pretreatment could alleviate salt stress-triggered ROS in various plant species, yet the involvement and molecular studies of melatonin-mediated salt tolerance in okra were severely impeded due to no reference genomic and transcriptomic data available for *A. esculentus*.

Due to the complex genetic background of okra still unknown to a large extent, the research of okra is severely hindered. Currently, the hybrid sequencing of combining NGS and SMRT is increasing, which can provide high-quality and complete transcriptome assembly, especially for species with sequencing genome [29, 36, 37]. In this study, more complete transcriptome of okra was generated by using the combination of Illumina sequencing and the long-read SMRT sequencing techniques, and our study provides the first comprehensive set of full-length isoforms in okra. A total of 8.5 Gb clean data was generated by SMRT transcriptome, including 221,014 FLNC reads and 121,360 non-redundant consensus isoforms (Additional file 2: Table S1). Based on sequence alignment in NR database, the okra isoforms had the highest homology with cotton (Fig. 5c), which is consistent with their genetic relations in evolution, both of them belonging to the family Malvaceae.

In the present study, the mitigation effects of melatonin in okra response to salt-induced inhibition were observed, as documented by plant resistant phenotype (Fig. 1), as well as by the higher levels of the net photosynthetic rate and chlorophyll content, and the scavenging of ROS of melatonin-pretreated plants compared with nontreated salt-stressed plants (Figs. 2, 3). The combination of Illumina sequencing and SMRT identified 1776, 1063 and 474 DEGs in okra seedlings treatment by NaCl, MT and NaCl+MT, respectively (Fig. 6). GO enrichment analysis of these genes suggested that the cellular and molecular events were involved in melatonin-induced salt resistance in okra (Fig. 7).

The modulation of metabolic homeostasis was also involved in the promotion of melatonin-mediated abiotic stress tolerance [16, 18]. In the study, the DEGs involved in multiple metabolic pathways that were significantly enriched, including nitrogen metabolism, sulfur metabolism, pentose and glucuronate interconversions, starch and sucrose metabolism, glutathione metabolism and alanine, aspartate and glutamate metabolism (Fig. 8). Among these pathways, carbohydrates and multiple amino acid metabolisms are important events in response to salt stress for osmotic adaptation [38]. Nitrogen metabolism, sulfur metabolism and alanine, aspartate and glutamate metabolism were significantly enriched in all three treatments. Previously, we performed a series of studies on okra, and analyzed the synthesis genes of okra bioactive constituents by next-generation sequencing, and explored the changes in the proteome level of okra under salt stress [39, 40]. The differentially expressed proteins (DEPs) were strongly associated with the biological processes of metabolism and response to stress. These results showed that the major reorientation of amino acid and carbohydrate and nitrogen metabolism may participate in the underlying mechanisms of melatonin in salt stress. Therefore, metabolic adjustments including multiple sugars, organic acids and amino acids provided beneficial effects for melatonin-treated plants respond to salt stress. Moreover, plant hormone signal transduction and inositol phosphate metabolism were only two pathways enriched in MT + NaCl treatment. Melatonin extensively involves in the metabolism of various plant hormones, such as ethylene, cytokinins, gibberellic acids, IAA, and abscisic acid [41]. DEPs associated with salt signalling were also identified by proteomic analysis under salt stress. Under salt stress conditions, several halotolerant plants accumulated high-levels of inositol [42, 43].

There were eight pathways significantly enriched only in melatonin-treated okra plants, including cysteine and methionine metabolism, carbon fixation photosynthetic organisms, diterpenoid biosynthesis, protein export, pyruvate metabolism, glutathione metabolism, plant-pathogen interaction, and protein processing in endoplasmic reticulum. These pathways might play important roles in melatonin-mediated resistance to salt stress in okra, indicating the widespread effects of melatonin. The biosynthesis of melatonin in plants is involved in several enzymes [44], including tryptophan decarboxylase (TDC), tryptamine 5-hydroxylase (T5H), caffeic acid O-methyltransferase (COMT), serotonin N-acetyltransferases (SNAT) and N-acetylserotonin-O-methyltransferases (ASMT). SNATs and ASMTs are considered as the rate-limiting enzymes of melatonin biosynthesis. Chloroplasts and mitochondria have been well documented the main sites of melatonin biosynthesis [45, 46]. These two organelles are more susceptible to oxidative stress than other cellular structures, thus producing more ROS. Hence melatonin biosynthesis in both organelles can easily provide protective effects for plants [44]. In addition, chloroplasts are the main site of photosynthesis in plants. Our previous proteomic analysis showed that there were 317 DEPs and they mainly located in chloroplast (113 proteins). There were 13 DEPs associated with ‘Porphyrin and chlorophyll metabolism’, which was consistent with transcriptome analysis under NaCl. Furthermore, the enrichment of “carbon fixation photosynthetic organisms” -related genes in melatonin-pretreated plants was in line with the effects of melatonin on enhancement of the photosynthesis and chlorophyll content in okra under salt stress, which might be probably due to the scavenging of ROS by melatonin.

A variety of TFs have been reported to play important roles in plant stress responses, which recognize DNA in a sequence-specific manner to regulate stress-responsive gene expression. These TFs include ethylene-responsive transcription factor, bZIPs, MYBs, NACs, WRKYs and so on. To date, some TFs have been well documented to be involved in melatonin-mediated stress tolerance. In *Arabidopsis*, the zinc finger of *Arabidopsis thaliana* 6 (ZAT6), a cysteine2/histidine2-type zinc finger TF, was involved in melatonin-mediated freezing stress resistance via activating CBF pathway [47]. Recently, it was found that MeWRKY79 and heat shock transcription factor 20 (MeHsf20) upregulates melatonin biosynthesis by binding to the N-acetylserotonin O-methyltransferase 2 (*MeASMT2*) promoter to confer the disease tolerance against cassava bacterial blight to cassava (*Manihot esculenta*) [48]. In tomato, the transcription factor heat shock factor A1a (HsfA1a) targeted the HSE in the promoter of the melatonin biosynthetic gene *COMT1* and activated the transcriptional expression of *COMT1* gene, thus increasing the accumulation of melatonin, which further improved the resistance of tomato plants to cadmium (Cd) [49]. In this study, various TFs were significantly differentially expressed by different treatments, such as bHLH, WRKY, NAC, and MYB. Most of them were up-regulated when treated with NaCl and down-regulated in melatonin-treated samples. This is consistent with the observations of some publications that multiple TFs were involved in melatonin-induced resistance to various stress [16, 17]. Many crucial biological processes depend on the regulation of TFs. These results indicate that TFs might contribute to improving salt stress resistance of melatonin-pretreated okra plants.

## Conclusions

Taken together, this study provides the first set of full-length isoforms in okra, and evidence for the protective roles of melatonin in okra against salt stress. This may be attributed to the systemic transcriptional regulation of stress-related genes, modulation of metabolic homeostasis, and the activation of antioxidants.

## Methods

### Plant materials, growth conditions and treatments

Seeds of okra *(Abelmoschus esculentus* L.) “xian zhi” were purchased from Vegetable Research Institute of Zhejiang Academy of Agricultural Sciences. The seeds were germinated in 3-cm-diameter black plastic pots filled with a mixture of vermiculite, peat, and perlite (1:2:1, v:v:v) and grown in a growth chamber with a 14/10-h photoperiod under a light intensity of 300 μmol m^− 2^ s^− 1^ at 28/24 °C day/night temperatures, and at 60% RH. All seedlings were uniformly watered every 2 days and fertilized weekly with 1/2 strength Hoagland’s solution [50]. When the two cotyledons were fully expanded, seedlings of uniform size were transferred to larger plastic pots (7-cm-diameter).

To determine the effects of exogenous application of melatonin in salt stress tolerance of plant, seedlings (with a true leaf and a newly unfolded young leaf) were first irrigated with 0, 50, or 100 μM melatonin (50 mL per plant) on roots for a total of three times (once every other day). Melatonin was dissolved by alcohol, and then diluted with Milli-Q water. Six days later, the plants were irrigated with 300 mM NaCl (50 mL per plant). Seven days later, samples of leaf (the second true leaf beneath the growing point) were harvested for biochemical assay after measuring physiological parameters. Harvested samples were rapidly frozen in liquid nitrogen and stored at − 80 °C until the biochemical measurements [50]. A 50 μM concentration of melatonin was used in the following experiments.

### Growth parameters

On day 7 of the salt treatment, the second true leaf length of seedlings was measured. Afterward, the all leaves of each plant were harvested and the leaf fresh weight was recorded.

### Determination of gas exchange and chlorophyll fluorescence

The net photosynthetic rate (Pn) of the second true leaf was measured using a Li-6400 portable photosynthesis system (Licor-6400, LICOR Inc., Lincoln NE, USA) with a red/blue light source according to the manufacturer’s instructions. The photosynthetic photon flux density (PPFD) and the temperature and external CO_2_ concentration was set at 1000 μmol m^− 2^ s^− 1^, 30 °C and 400 μmol mol^− 1^, respectively.

The maximum photochemical efficiency of photosystem II (PSII) (Fv/Fm) was measured with a Dual-PAM 100 Chl fluorescence analyzer (Heinz Walz, Effeltrich, Germany) after a 30 min dark-adaptation following the method of Wang et al. [51]. The Fv/Fm images were detected by Chlorophyll fluorescence Imager (CF Imager) (Technologica, United Kingdom, http://www.technologica. co.uk/).

### Measurement of chlorophyll content

Total chlorophyll from the second true leaf was extracted in 80% (v/v) acetone in darkness for 24 h, and then calculated by determining the absorbance at 663 nm and 645 nm.

### Determination of malondialdehyde, H_2_O_2_, O_2_^·—^ and anti-oxidant enzymes activities

The malondialdehyde (MDA) and H_2_O_2_ contents, and GR activity were measured with the assay kits of Jiangsu Keming Biotechnology Institute (Suzhou, China). For the determination of MDA content and GR activity, 0.1 g of leaf tissues were ground to powder with 1 ml buffer I [50 mM phosphate buffer (pH 7.8), containing 0.5% (w/v) Triton-100, 0.1 mM EDTA, and 2% PVP], centrifuged at 10,000 rpm for 20 min at 4 °C and then the supernatant was used for determination according to the manufacturer’s protocol. For the determination of H_2_O_2_ contents, 1 ml acetone replaced the extraction buffer I.

The production rate of O_2_^·—^ was measured according to the method described by Elstner and Heupel [52]. In brief, about 0.1 g leaf was homogenized with 3 mL 65 mM potassium phosphate buffer (PBS) (pH 7.8), and centrifuged at 10,000 rpm at 4 °C for 20 min. Then, 0.5 mL supernatant was mixed with 0.1 mL 10 mM hydroxylamine hydrochloride and 0.5 mL PBS and incubated at 25 °C for 20 min. Subsequently, 1 mL 7 mM α-naphthylamine and 1 mL 58 mM sulfonamide were added to the incubation mixture and incubated at 25 °C for another 20 min. Then, 3 mL chloroform was added and centrifuged 10,000 rpm for 5 min. The absorbance was measured at 530 nm.

For the measurement of POD, CAT and SOD activities, about 0.2 g of leaf tissues were homogenized in 50 mM phosphate buffer solution (pH 7.8). The extracts were centrifuged at 10,000 rpm at 4 °C for 20 min. Supernatants were collected for enzymes activities analysis as described by Hou et al. [53].

### RNA preparation and assessment of quality

All the samples were grinded on liquid nitrogen and the total RNA was extracted by TRIzol reagent and DNA was removed by DNase I (Takara). RNA purity (OD260/280) was measured using Nanodrop2000 (ThermoFisher, Waltham, MA, USA). RNA integrity was checked using the Agilent Bioanalyzer 2100 system (Agilent Technologies, CA, USA).

### PacBio library preparation, sequencing and data analysis

RNA at OD260/280 > 2.0, OD260/230 at 1.8–2.1 and integrity number > 8 was selected for subsequent studies. The Iso-Seq library was prepared using the Clontech SMARTer PCR cDNA Synthesis Kit and the BluePippin Size Selection System protocol as described by Pacific Biosciences (PN 100–092–800-03). Sequencing was performed on a Pacbio Sequel instrument. Sequence data were processed using the SMRTlink 5.1 software. The subreads sequence was obtained by the correction between subreads. According to whether the sequence contained 5’end primer, 3’end primer and polyA tail, the sequences were divided into full-length sequence and non-full-length sequence. Isoform level clustering was used to cluster the full-length sequence to obtain the Cluster consensus sequence; finally, the non-full-length sequence was used for polishing the obtained consensus sequence to obtain high-quality sequences for subsequent analysis [54]. Any redundancy in corrected consensus reads was removed by CD-HITv4.6 package (−c 0.95 -T 6 -G 0 -aL 0.00 -aS 0.99) to obtain final transcripts for the subsequent analysis.

### Illumina library preparation, sequencing and data analysis

A total amount of 3 μg RNA per sample was used for the library preparation [25]. The NEBNext® Ultra™ RNA Library Prep Kit for Illumina® (NEB, USA) was used to generate sequencing libraries as described by the manufacturer. The mRNA was enriched by Oligo (dT) magnetic beads and the mRNA is randomly interrupted. Using fragmented mRNA as a template, the first strand of cDNA was synthesized in the M-MuLV reverse transcriptase system, and the second strand of cDNA was synthesized using DNA polymerase I. The purified double stranded cDNA was repaired at the end, “A”tail was added and connected to the sequencing adaptor. The cDNA of about 250-300 bp was screened by AMPure XP beads and amplified by PCR. The PCR product was purified by AMPure XP beads again, and finally the library was obtained. The libraries were sequenced on an Illumina Hiseq X ten platform and paired-end reads were generated. Clean reads were obtained by removing reads containing adapter, reads containing ploy-N and low quality reads from raw data. Fragments per kilobase of transcripts per million mapped reads (FPKM) was used to estimate the gene expression level. Differential expression analysis was performed using the DESeq R package (1.10.1). Genes with an adjusted *P*-value < 0.05 found by DESeq were assigned as differentially expressed [25].

### Functional annotation of unigenes

Transcripts were annotated by searching against seven public databases [37]: NR (NCBI non-redundant protein sequences); NT (NCBI non-redundant nucleotide sequences); Pfam (Protein family); KOG (Clusters of Orthologous Groups of proteins); Swiss-Prot (A manually annotated and reviewed protein sequence database); KO (KEGG Ortholog database) [55]; GO (Gene Ontology).

GO enrichment analysis of DEGs were implemented by the GOseq R package GO terms with corrected P<0.05 were considered significantly enriched. KOBAS software was used to test the statistical enrichment of DEGs in KEGG pathways.

### Quantitative RT-PCR analysis

Quantitative RT-PCR assay was performed using the SYBR® Premix Ex TaqTM kit (TaKaRa, Japan) on a Roche LightCycler480 instrument [53]. An endogenous okra *actin* gene was selected as an internal reference gene. Primers used in the experiment are listed in Additional file 8: Table S3.

### Statistical analysis

All data were subjected to one-way analysis of variance (ANOVA) with Duncan’s multiple range test at *P* < 0.05 using SPSS 16.0 (SPSS Inc., Chicago, IL, USA). All figures were expressed as mean ± standard deviation (SD) of three replicates.

## Supplementary Information

**Additional file 1: Figure S1.** Phenotype traits of okra seedlings exposed to salt stress for 7 d by irrigating with different concentrations of NaCl solution.

**Additional file 2: Table S1.** PacBio Iso-seq output statistics.

**Additional file 3: Figure S2.** Functional classification of unigenes by KOG analysis.

**Additional file 4: Table S2.** The KEGG pathways of isoforms.

**Additional file 5: Figure S3.** Functional annotation of unigenes by GO analysis.

**Additional file 6: Figure S4.** Heatmap of Pearson’s correlation between samples.

**Additional file 7: Figure S5.** Numbers of the primary transcription factor (TF) families.

**Additional file 8: Table S3.** Primers for quantitative real-time (qRT) PCR.

## Data Availability

All of the transcriptional data were deposited in the NCBI Sequence Read Archive (BioProject for PacBio Iso-Seq sequencing: PRJNA685320, https://submit.ncbi.nlm.nih.gov/subs/sra/SUB8747384/overview; BioProject for Illumina sequencing: PRJNA668637, https://submit.ncbi.nlm.nih.gov/subs/sra/SUB8322037/overview). All data generated or analysed during this study are included in this published article and its supplementary information files.

## Abbreviations

ROS: Reactive oxygen species
POD: Peroxidase
CAT: Catalase
SOD: Superoxide dismutase
CCS: Circular consensus sequence
Flnc: Full-length non-chimeric
ICE: Iterative clustering for error correction
DEG: Differential expression gene
TF: Transcription factors
