# Supplementary material for: Comparative physiological and full-length transcriptome analyses reveal the molecular mechanism of melatonin-mediated salt tolerance in okra (Abelmoschus esculentus L.)
Source: BMC Plant Biol. 2021 Apr 15;21:180. doi: 10.1186/s12870-021-02957-z (PMC8051126; doi:10.1186/s12870-021-02957-z)
Supplement: Supplementary file 2 — Additional file 2: Table S1. PacBio Iso-seq output statistics. [file 12870_2021_2957_MOESM2_ESM.docx]

**Additional file 2** of Comparative physiological and full-length transcriptome analyses reveal the molecular mechanism of melatonin-mediated salt tolerance in okra (*Abelmoschus esculentus* L.) (Yihua Zhan, Tingting Wu, Xuan Zhao, Zhanqi Wang, Yue Chen)

**Additional file 2: Table S1** PacBio Iso-seq output statistics

| Subreads base(G) | Subreads number | Average subreads length | N50 | Number of Circular Consensus Sequence | Number of five prime reads | Number of three prime reads | number of poly-A reads | number of full-length reads | number of full-length non-chimeric reads | Average flnc read length |
| --- | --- | --- | --- | --- | --- | --- | --- | --- | --- | --- |
| 8.54 | 3644038 | 2345 | 2715 | 286218 | 261100 | 264446 | 257198 | 234728 | 221014 | 2569 |
