# Supplementary material for: Comparative physiological and full-length transcriptome analyses reveal the molecular mechanism of melatonin-mediated salt tolerance in okra (Abelmoschus esculentus L.)
Source: BMC Plant Biol. 2021 Apr 15;21:180. doi: 10.1186/s12870-021-02957-z (PMC8051126; doi:10.1186/s12870-021-02957-z)
Supplement: Supplementary file 3 — Additional file 3: Figure S2. Functional classification of unigenes by KOG analysis. [file 12870_2021_2957_MOESM3_ESM.docx]

**Additional file 3** of Comparative physiological and full-length transcriptome analyses reveal the molecular mechanism of melatonin-mediated salt tolerance in okra (*Abelmoschus esculentus* L.) (Yihua Zhan, Tingting Wu, Xuan Zhao, Zhanqi Wang, Yue Chen)


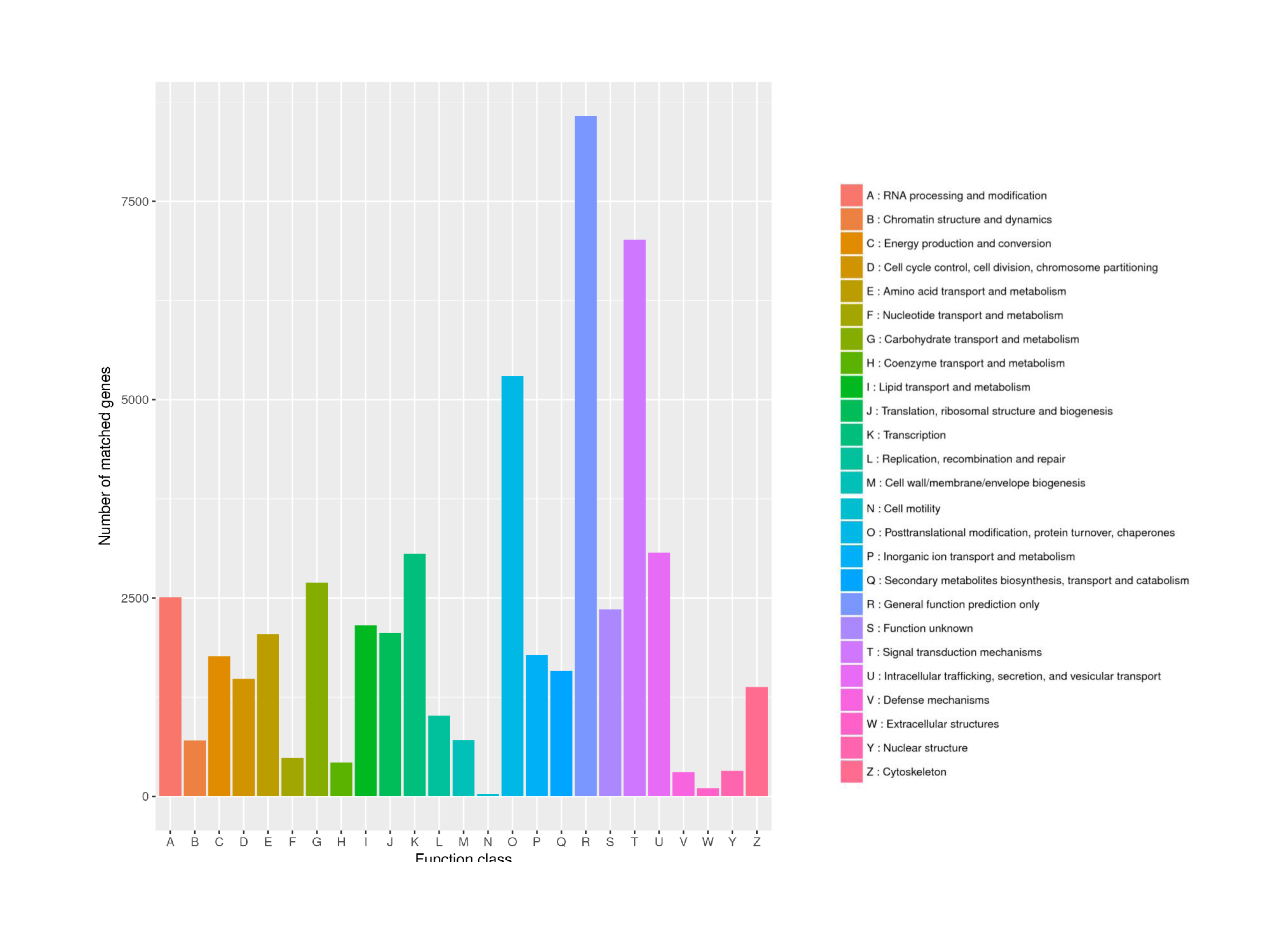


**Fig. S2** Functional classification of unigenes by KOG analysis.
