# Supplementary material for: Comparative physiological and full-length transcriptome analyses reveal the molecular mechanism of melatonin-mediated salt tolerance in okra (Abelmoschus esculentus L.)
Source: BMC Plant Biol. 2021 Apr 15;21:180. doi: 10.1186/s12870-021-02957-z (PMC8051126; doi:10.1186/s12870-021-02957-z)
Supplement: Supplementary file 8 — Additional file 8: Table S3. Primers for quantitative real-time (qRT) PCR. [file 12870_2021_2957_MOESM8_ESM.docx]

**Additional file 8** of Comparative physiological and full-length transcriptome analyses reveal the molecular mechanism of melatonin-mediated salt tolerance in okra (*Abelmoschus esculentus* L.) (Yihua Zhan, Tingting Wu, Xuan Zhao, Zhanqi Wang, Yue Chen)

**Additional file 8: Table S3** Primers for quantitative real-time (qRT) PCR

| Gene | Gene accession | Primers sequence (from 5′ to 3′) |
| --- | --- | --- |
| *Actin*  *Myb44*  *Myb1R1*  *NAC83*  *NAC82*  *WRKY70*  *WRKY46*  *WRKY20*  *WRKY25*  *WRKY32* | i2_HQ_OkraNGS_c92341/f5p2/2106  i2_LQ_OkraNGS_c66290/f1p6/2807  i1_LQ_OkraNGS_c40250/f1p2/1378  i2_LQ_OkraNGS_c8741/f1p2/2376  i2_LQ_OkraNGS_c82352/f1p0/2472  i1_LQ_OkraNGS_c46834/f1p0/1071  i1_LQ_OkraNGS_c32436/f1p5/1255  i3_LQ_OkraNGS_c23822/f1p0/3206  i2_LQ_OkraNGS_c88921/f1p4/2034  i2_HQ_OkraNGS_c35965/f2p0/2748 | F: 5'- TTGTATTGCTCGACGTACCAA-3'  R: 5'- TTATACCCATCAACACGTCCC-3'  F: 5'- CATCTTGGACGCTCATGCT-3'  R: 5'- TCCCAATGCACAATGATCACC-3'  F: 5'- TTGCAACTTACCCCGAGGAC-3'  R: 5'- TTACTGGCTCCTTCGTCACC-3'  F: 5'- AAGAACCAAACTCATAGCCAT-3'  R: 5'- CTGCTTTCTTCGGAGTCGTC-3'  F: 5'- CTGAAGCTCCCATGATTGTCG-3'  R: 5'- TCATCTTCCTCGGAGACGTT-3'  F: 5'- AAACACTGTCGAGCAATGAGA-3'  R: 5'- GTCTCTTCTTACAACAAGGGG-3'  F: 5'- TCTCAATTCGCCGCTACGTT-3'  R: 5'- GTTTTAGCCTTAAGCCCGGAT-3'  F: 5'- CCAGCTGTTTTGAGTTTAGACC-3'  R: 5'- TCAGCAGGAACACTAGTCACA-3'  F: 5'- CATTCTGCCATCTCCTACCAC-3'  R: 5'- CTGCATCTGATTGTACTCGG-3'  F: 5'- CTGTACCAAAGAAGCGACA-3'  R: 5'- TAGCTGACTCCATTGCCTT-3' |
